# Challenges of Diagnosing Severe Ehrlichiosis in Orthotopic Liver Transplant Recipients

**DOI:** 10.1155/2021/8285326

**Published:** 2021-11-17

**Authors:** Melissa Parkinson, Spandana Vuyyuru, Jay Patel, Chinelo Animalu

**Affiliations:** ^1^Internal Medicine and Pediatrics Residency Program, University of Tennessee Health Science Center, Memphis, USA; ^2^College of Medicine, University of Tennessee Health Science Center, Memphis, USA; ^3^Division of Infectious Diseases, University of Tennessee Health Science Center, Memphis, USA

## Abstract

In recent solid organ transplant recipients, acute febrile illness is usually a source of grave concern and a diagnostic dilemma, especially if no response is noted after initiation of broad antimicrobial therapy. Human Monocytic Ehrlichiosis (HME) is a tick-borne illness caused by *Ehrlichia chaffeensis* and is not considered an opportunistic infection in immunocompromised patients such as solid organ transplant patients. Ehrlichiosis in immunocompromised patients can be life-threatening, and a strong index of suspicion is needed, especially in patients who live in endemic areas, for proper treatment initiation with doxycycline. We report a case of a 40-year-old male who received an orthotopic liver transplant six months earlier secondary to primary sclerosing cholangitis, on chronic immunosuppressive medication, who presented with complaints of sudden onset fever associated with nausea, vomiting, and diarrhea. Initial extensive infectious workup was negative and no response to empiric antimicrobials. There was suspicion for ehrlichiosis prompting empiric doxycycline use. Subsequently, *E. chaffeensis* polymerase chain reaction (PCR) was positive, and the antibiotic regimen was de-escalated to only doxycycline with complete resolution of his symptoms and progressive improvement in previously abnormal biochemical indices.

## 1. Background and Introduction

Solid organ transplant is a life-saving treatment for many patients with end-stage organ disease. However, the immunosuppressive regimens these patients require leave them susceptible to severe infections from both opportunistic and common organisms. The wide range of potential infectious agents and blunted immune response in these patients can make prompt diagnosis and treatment difficult.

**Figure 1:**
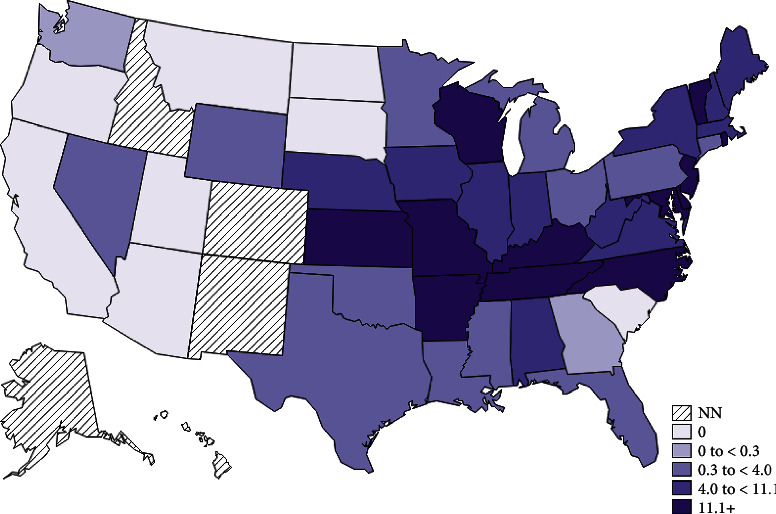
Annual incidence (per million population) of HME in the United States in 2019. This graph shows the general distribution and incidence of HME in the United States, with most cases occurring in the south-central and southeastern United States. Courtesy of the Centers for Disease Control and Prevention [1].

**Figure 2:**
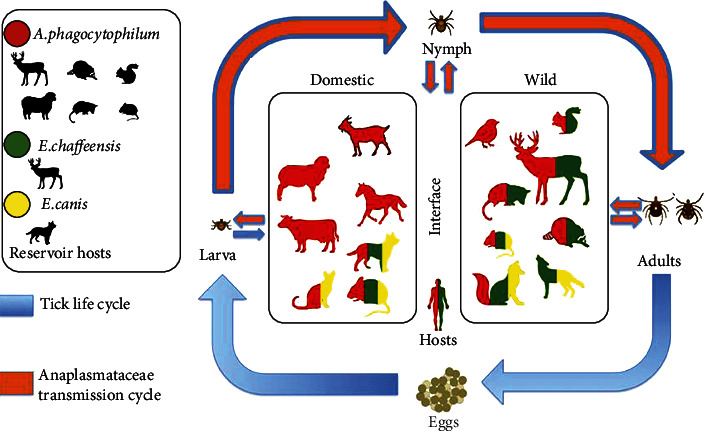
Tick-borne pathogen transmission. This image shows the life cycle of Ehrlichia species and which animal different tick-borne pathogens can inhabit. This also shows the different ways humans can come in contact with ticks. Image courtesy of the open access article by Rojero-Vázquez et al. [2].

Ehrlichiosis is a disease caused by obligate intracellular bacteria from the genus *Ehrlichia* transmitted via tick bite that infects human and animal leukocytes. *E. chaffeensis* is the most common species to infect humans and causes Human Monocytic Ehrlichiosis (HME). Rates of reported HME rose from just 200 in 2000 to 1,799 in 2018 and continue to rise, making it an increasingly important illness [1]. *E. chaffeensis* is transmitted primarily through the lone star tick and is endemic (Figure 1 [1]) to the southeastern, south-central, and mid-Atlantic United States [1]. Contact with the ticks and subsequent transmission can occur in areas where humans are with natural tick reservoirs (e.g., cats and deer) (Figure 2 [2]).

Manifestation of HME can range from asymptomatic to severe, with severe illness being more likely in the elderly, those with comorbid conditions, and immunocompromised patients. Many of the common early symptoms are nonspecific, including fever, chills, malaise, myalgia, headache, nausea, vomiting, diarrhea, and loss of appetite. Rash and neurological symptoms like confusion may also be present [1]. Delays in treatment and immunocompromised states can lead to more severe late disease with meningoencephalitis, respiratory failure, uncontrolled bleeding, organ failure, and death [1]. Definitive diagnosis can be made with several laboratory techniques, including serology, polymerase chain reaction (PCR), and buffy coat examination. Treatment with doxycycline is usually initiated based on a presumptive diagnosis when clinical suspicion is high. However, when tick-borne illness is not suspected early on, definitive treatment can be critically delayed due to the initial empiric antibiotic regimen's exclusion of *E. chaffeensis* coverage.

## 2. Case Report

We present the case of a 40-year-old male who six months prior underwent an orthotopic liver transplant secondary to primary sclerosing cholangitis on chronic immunosuppressive therapy. He presented to the emergency room with complaints of sudden onset fever ongoing for three days associated with nausea, vomiting, and diarrhea (about five to six watery bowel movements per day). He also endorsed fatigue. He denied chest pain, shortness of breath, headache, confusion, or vision changes. His immunosuppressive medications consisted of prednisone, mycophenolate mofetil, tacrolimus, and valganciclovir. He previously had cytomegalovirus (CMV) viremia, for which he was treated with valganciclovir. Serial CMV PCR was negative, and the valganciclovir dose was decreased to a maintenance dose.

At the time of initial presentation, he was febrile with a maximum temperature of 38.9°C, blood pressure 93/55 mmHg, pulse 101 beats/min, and oxygen saturation 98% on room air. He was alert, oriented, cooperative, and in no apparent distress. The physical exam was unremarkable, with no rashes or lesions noted.

Initial labs revealed sodium of 130 mEq/L, potassium 5.6 mEq/L, chloride 105 mEq/L, creatinine 1.60 mg/dL, albumin 4.6 g/dL, total bilirubin 1.2 mg/dL, AST 44 U/L, ALT 51 U/L, WBC 1.70 thou/mcL with absolute neutrophil count of 0.6 thou/mcL, platelets 40 thou/mcL, and hemoglobin 8.7 g/dL. CMV PCR was negative. Blood cultures were sent and were subsequently negative. A chest X-ray did not reveal any acute abnormalities. Abdominal ultrasound showed findings consistent with medical renal disease only. Gastrointestinal PCR panel was negative for enterotoxigenic E. coli, Giardia, Norovirus, Salmonella, Shiga-like Toxin producing E. coli, Plesiomonas shigelloides, Vibrio, Yersinia enterocolitica, enteroaggregative E. coli, Cyclospora cayetanensis, Entamoeba histolytica, Adenovirus F 40/41, Astrovirus, Sapovirus, Enteropathogenic E. coli, and Shigella/Enteroinvasive E. coli. Clostridium difficile and SARS-CoV-2 PCR were negative. Serum Cryptococcus neoformans and histoplasmosis antigens were negative. Acute hepatitis A, B, and C panel was negative. Rocky Mountain spotted fever IgM titer and Rickettsia typhi IgM and IgG antibodies were negative. Urinalysis was unremarkable.

**Figure 3:**
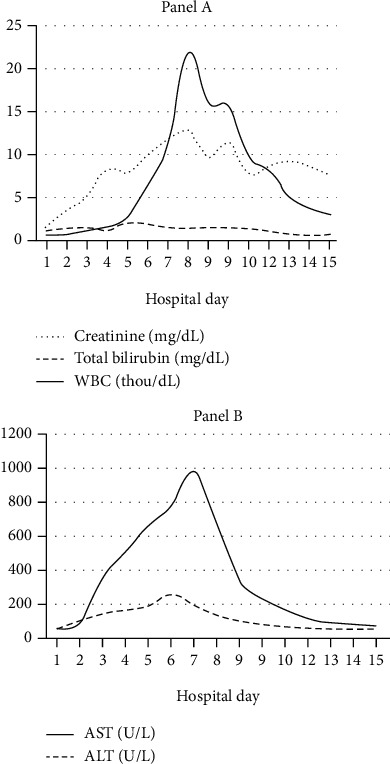
Trends of key lab findings throughout hospital stay. Doxycycline was started on hospital day 3 for suspected ehrlichiosis. The patient had been started on hemodialysis due to acute renal failure on hospital day 4 and was taken off on day 10 due to marked clinical improvement. The progression of creatinine (mg/dL), WBC count (thou/mcL), and total bilirubin (mg/dL) over his hospital stay is seen in (a). The progression of AST (U/L) and ALT (U/L) over his hospital stay is seen in (b).

He was started on empiric vancomycin, meropenem, and micafungin (doses adjusted for renal function) without any clinical improvement in 48 hours. His immunosuppressive medication doses were reduced appropriately. His fever persisted, and renal function, as well as liver functions, declined rapidly (Figure 3), prompting an infectious disease consult. On further questioning, the patient revealed he worked as a resource manager in a state park and is exposed regularly to ticks; he did not recall any obvious tick bites and had never had to pluck out a tick from his skin. He denied eating unpasteurized goods and did not consume uncooked meat or fish. He had not been swimming in public pools.

Given this history, intravenous doxycycline for presumptive ehrlichiosis was initiated on hospital day 3 while awaiting studies. Subsequently, *E. chaffeensis* PCR returned positive. He required a short ICU stay for vasopressor support due to septic shock. Given his severe acute kidney injury and creatinine clearance decline to <15 mL/min, hemodialysis was initiated on hospital day 4. His immunosuppressive medication regimen was initially held on admission and later changed to prednisone and tacrolimus, while mycophenolate was discontinued.

After initiation of doxycycline, clinical improvement was noted both in symptoms and laboratory indices (Figure 3). He completed a 10-day course of doxycycline and was taken off hemodialysis on hospital day 10. The patient was discharged home in a stable condition and, since his discharge, has continued to do well.

## 3. Discussion

Since its first recognition in the late 1980s, cases of HME have been rising, with over 1000 cases reported by the CDC annually since 2012, occurring in both immunocompromised and immunocompetent patients [1]. Case numbers in 2018 were over eight times higher than in 2000, making HME important to consider when a patient presents with nonspecific findings, particularly in an endemic area [1]. While many studies examine HME in the context of the United States, there has been an increasing incidence of HME internationally on continents such as Asia and Africa [3]. Various studies are emerging that have shown *Ehrlichia* species found in different animal vectors that were thought not to host them, suggesting that there could be more unknown reservoirs of this bacterium and that overall distribution and disease of this bacterium may be more widespread than previously thought [4–6].

The number of HME cases occurring in solid-organ transplant (SOT) recipients makes up a small but significant portion of overall cases. It is important to recognize this as a potential cause of infection in SOT patients, particularly in endemic areas. A literature search was done, and all known cases of *Ehrlichia* complicating various SOT are tabulated in Table 1. There were 143 found in the literature, and, interestingly, doxycycline was used in the vast majority of these patients, and most patients survived as well.

Diagnosis of ehrlichiosis in transplant recipients can often be challenging due to the low prevalence of solid-organ transplant (SOT) HME, nonspecific symptoms, which can be seen in a wide range of infection as well as organ rejection, and blunting of the immune response [3, 4]. Common symptoms reported from *Ehrlichia* infections in transplant patients from one review of cases include a history of fatigue (30%), subjective fevers (95-100%), headache (55-60%), and GI symptoms (40-60%), which were similar across other reports [7–11]. Our patient presented with fever, diarrhea, nausea, vomiting, and fatigue—similar symptoms to those reported in other SOT patients; however, he had no headache or other neurologic symptoms. Less commonly, HME can present with cutaneous manifestations, pulmonary embolism, severe disease leading to hemophagocytic lymphohistiocytosis, and fulminant organ failure [12].

Preemptive treatment is usually initiated for suspected ehrlichiosis while workup for definitive diagnosis is in progress. Several current diagnostic methods include serologies, peripheral blood smear (buffy-coat preparation) examination, PCR, immunohistochemical (IHC) staining, and culture. Serologic studies can be done via an indirect fluorescent antibody test (IFA) or enzyme-linked immunosorbent assay (ELISA). IFA is the standard reference test for ehrlichiosis; it is widely available and can be obtained through state health departments [13]. This assay requires comparing antibody titers of acute and convalescent serum samples collected 2–4 weeks apart to demonstrate evidence of a fourfold seroconversion. While the test is 94-100% sensitive, antibody titers are usually low during the first week of illness, decreasing utility in detecting HME in the early phase [13–15]. Thus, IFA is infrequently due to minimal early clinical utility and time-consuming nature [16]. In addition, ELISA also measures antibody titers and is excellent for confirming findings from IFA, but it is not very good at evaluating variations in antibody titers with time [13, 16]. Thus, similar to IFA, ELISA's clinical utility is low and better suited for clinical trials [16]. Peripheral blood smear examination using a buffy-coat preparation is usually useful within the first week of illness, where microscopic examination may reveal a collection of microcolonies of *Ehrlichia* in the cytoplasm of white blood cells [13]. The utility of examining peripheral blood depends on the patient population [17]. Sensitivity of doing a peripheral blood smear in immunocompetent people is 17%; however, immunocompromised patients can have a sensitivity as high as 100%, increasing utility in the transplant population [17, 18]. This is likely due to the immunocompromised state enabling a more considerable morula burden, increasing the chance of seeing morula on slides. PCR is a rapid diagnostic test that is becoming more widely available, and it has good sensitivity (85%) and specificity (100%) [14, 19]. It is most sensitive within the first week of symptom onset, and a positive result is considered diagnostic; however, a negative result does not rule out ehrlichiosis, especially if antibiotics have begun [20]. IHC staining of ehrlichial antigens in tissue can be done, but this is not widely available and not routinely used for acute diagnosis, limiting clinical utility [20]. These can be performed in bone marrow, live tissue specimens, or postmortem specimens [20]. Lastly, culture is slow, not widely available, and is not very useful [16, 20].

This case highlights the importance of taking a thorough history to identify potential exposures. Treatment for HME was only initiated after a thorough exposure history revealed that the patient worked outside and had significant tick exposure. Doxycycline is the drug of choice for the treatment of HME [21]. However, in some patients with a severe allergy to doxycycline, alternative therapy such as rifampin and chloramphenicol has been used even though there are no robust studies available at this time supporting the routine use of these agents [22]. There are case reports where successful use of these agents has been documented; chloramphenicol is not readily available in the United States and is associated with hematological side effects [22]. Travel history to endemic areas, work and hobby history, and a thorough skin exam should all be performed when concerned for possible HME infection. While a definitive diagnosis of HME can be made using a variety of methods, including PCR and serology, there are diagnostic clues that can be identified in an initial lab workup that is typical of HME, including leukopenia, thrombocytopenia, elevated liver enzymes, elevated creatinine, and less commonly hyponatremia, pancytopenia, and decreased serum albumin levels [7–11]. In this case, the patient had lab findings consistent with HME, including hyponatremia, pancytopenia, elevated creatinine, and elevated liver enzymes (Figure 3).

Early detection of disease is crucial to prevent disease progression and end-organ damage. In one study, delayed time to treatment was associated with the need for ICU admission [8]. In this case, delays in the patient's diagnosis led to renal failure requiring hemodialysis and septic shock requiring vasopressor support. Immunosuppression has often been suggested as a possible risk factor for worse prognosis and ICU admission, but the data are mixed. One study found that immunosuppression was associated with decreased need for ICU admission, while others showed that it was associated with more severe disease [8, 10, 23]. Transplant type has been associated with worse outcomes and more prolonged ICU admissions, with lung transplant being associated with a poorer prognosis compared with other solid organ transplants [10, 24]. The case fatality rate has been reported between 0% and 26% for transplant patients, which is similar to the case fatality rate in all HME cases [8–10]. Case fatality has decreased since its discovery despite the number of cases increasing [1].

This case report adds to the body of literature on ehrlichiosis in transplant patients, serves as an example of clinical patterns of infection, and stresses the importance of early identification of disease to enable prompt treatment within the vulnerable population of transplant recipients.

## Figures and Tables

**Table 1:** All cases of ehrlichiosis complicating solid organ transplants.

Total cases	Age median (Yr, range)	Sex	Transplant type	Diagnosis method	Management	Outcome	References
25	54 (26-68)	68% M	H (2), Li (5), Lu (5), R (13)	PCR	Dox	S	Lawrence et al. [10]
51	57 (9-72)	75% M	H (12), Li (7), Lu (12), R (18), R/P (2)	PCR	NA	S	Otrock et al. [11]
1	69	F	Lu	PCR, PBS	Dox	S	Regunath et al. [18]
15	50 (15-73)	87% M	H (6), Li (1), Lu (1), R (7)	PCR (93%), Ser (7%)	Dox	S	Thomas et al.[9]
2	56, 57	50% M	R (2)	PCR (50%), Ser (50%)	Dox, Tigecycline	S	Sachdev et al. [25]
1	38	M	Lu	PCR, PBS	Dox	S	Safdar et al. [23]
1	57	M	Li	PCR, Ser	Dox	S	Liddell et al. [24]
1	63	M	R	PCR, PBS	Dox	S	Kumar et al. [26]
1	60	M	R	PCR, PBS, Ser	Dox	S	Cotant et al. [27]
1	11	M	R	PCR	Dox	S	Buller et al. [28]
1	NA	M	R	PCR	Dox	S	Sadikot et al. [29]
1	35	M	R	Ser, PCR	Dox	S	Schutze et al. [30]
1	47	M	Li	PCR	Dox	S	Tan et al. [14]
1	27	M	R	PBS, Ser	Dox	S	Dorn et al. [31]
1	35	F	R	PCR	Dox	S	Masterson et al. [7]
27	NA	NA	NA	PCR	Dox	NA	Kuriakose et al. [8]
1	51	M	Li	Ser	Dox	S	Antony et al. [32]
3	38, 41, 50	66% M	P/R, P/R, P	PBS	Dox	66% S	Trofe et al. [33]
1	67	M	R	PBS, PCR	Dox	S	Adachi et al. [34]
1	66	F	R	PBS, PCR	Dox	S	Vannorsdall et al. [35]
1	57	M	R	PCR	Dox	S	Hassan et al. [36]
5	43 (5-70)	M	Lu (1), R (4)	PCR (80%); PBS (20%)	Dox	60% S	Saha et al. [15]

D: death; Dox: doxycycline; F: female; H: heart; Li: liver; Lu: lung; M: male; NA: not available; P: pancreas; PBS: peripheral blood smear; PCR: polymerase chain reaction; R: renal; Ser: serology; S: survived; Yr: year. This table helps demonstrate that, while HME can occur in a wide range of ages, it is most often seen in older patients. Interestingly, PCR was most often used to help diagnose HME. Notably, most patients received doxycycline and had a good outcome.

## Data Availability

The data used to support the findings of this study are included within the article.
